# Leveraging Data Science to Understand Aging Trajectories

**DOI:** 10.1093/geroni/igaf122.1245

**Published:** 2025-12-31

**Authors:** Jonathan Wren

## Abstract

Aging is heterogeneous: individuals follow distinct cognitive, cardiometabolic, and neurodegenerative trajectories shaped by genetics, environments, and care contexts. This symposium showcases bioinformatics/data-science strategies for modeling that heterogeneity and extracting actionable signals for prevention and treatment. Talks will span (i) longitudinal and genetically informed models linking adversity, social determinants, and polygenic variation to cognitive aging; (ii) machine-learning methods for multimodal biomedical data (e.g., digital pathology and imaging), emphasizing interpretability and clinical utility; and (iii) translational insights from large, deeply phenotyped cohorts to improve risk stratification and trial enrichment in dementia.

